# Supplementary figures and images for: Comparison of IL-33 and IL-5 family mediated activation of human eosinophils
Source: PLoS One. 2019 Sep 6;14(9):e0217807. doi: 10.1371/journal.pone.0217807 (PMC6730854; doi:10.1371/journal.pone.0217807)

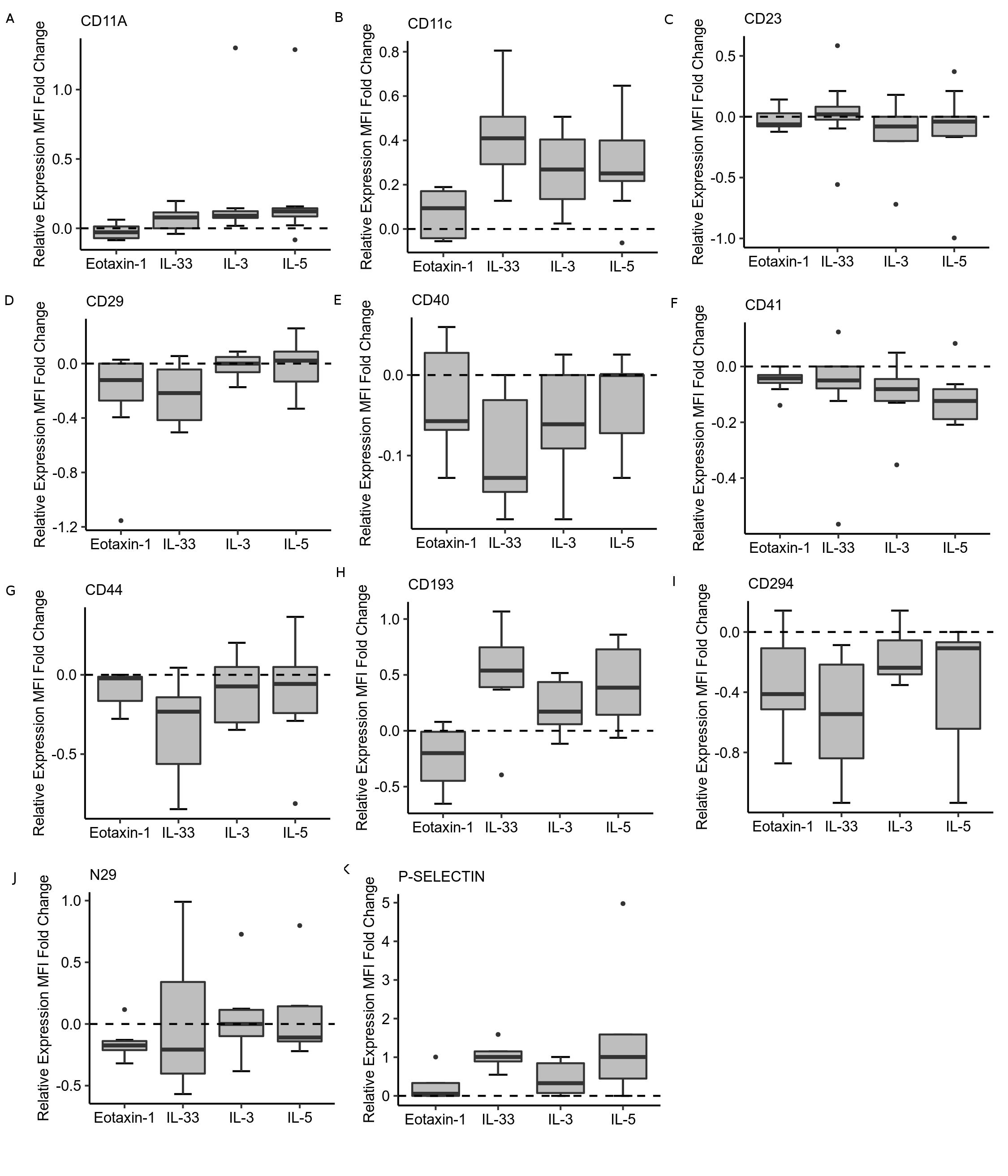

Supplement: S1 Fig — Box plots of normalized fold change for A) CD11a, B) CD11c, C) CD23, D) CD29, E) CD40, F) CD41, G) CD44, H) CD193, I) CD294, J) N29, K) CD62P after 4 hour stimulation with either IL-3, IL-5, IL-33, eotaxin-1 compared with unstimulated control, represented by dotted line (n = 7–8). These samples did not demonstrate significant difference when compared to unstimulated control. (TIF) [file pone.0217807.s001.tif]
